# Self-respect through ability to keep fear of frailty at a distance: Successful ageing from the perspective of community-dwelling older people

**DOI:** 10.3402/qhw.v8i0.20194

**Published:** 2013-03-18

**Authors:** Helena M. Hörder, Kerstin Frändin, Maria E. H. Larsson

**Affiliations:** 1 Neuropsychiatric Epidemiology Unit, Institute of Neuroscience and Physiology, Sahlgrenska Academy at Göteborg University, Göteborg, Sweden; 2 Division of Physiotherapy, Department of Neurobiology, Care Science and Society, Karolinska Institutet, Stockholm, Sweden; 3 Department of Clinical Neuroscience and Rehabilitation/Physiotherapy, Institute of Neuroscience and Physiology, Sahlgrenska Academy at Göteborg University, Göteborg, Sweden

**Keywords:** Age well, healthy ageing, qualitative research, quality of life, older persons, content analysis, experiences

## Abstract

With population ageing, there is an increased interest in how to promote a good old age. A predominant concept in these discussions is successful ageing, which is mainly based on researchers’ definitions. This article aims to explore successful ageing from the perspective of community-dwelling older people (24 persons aged 77–90 years). Individual open interviews were conducted and analysed according to qualitative content analysis. An overarching theme was formulated as “self-respect through ability to keep fear of frailty at a distance”. This embraced the content of four categories: “having sufficient bodily resources for security and opportunities”, “structures that promote security and opportunities”, “feeling valuable in relation to the outside world”, and “choosing gratitude instead of worries”. Ageing seems to be a dynamic process rather than a static structure and might therefore be susceptible to actions. Paying attention to attitudes and treating the older person with respect, particularly with regard to worries about increasing vulnerability, can lead to better ways of promoting successful ageing.

Although health and social welfare authorities worldwide are aiming to promote a good old age, the older persons’ own views of what this means are seldom heard. A concept that is frequently used in these discussions is successful ageing, which has been debated in gerontology since the early 1960s (Havighurst, 1961). The dramatic increase in life expectancy during the 20th century in many parts of the world (Christensen, Doblhammer, Rau, & Vaupel, 2009) has led to a further increased interest in this concept and its overlapping terms such as healthy ageing, active ageing, ageing well (Strawbridge, Wallhagen, & Cohen, 2002), and quality of life (Bowling, 2008). The most widely accepted definition of successful ageing so far is that proposed by Rowe and Kahn (1997); absence or avoidance of disease and risk factors for disease, maintenance of physical and cognitive functioning, and engagement with life. Baltes and Baltes (1990) introduced another prominent model focusing on a process with behavioural and psychological adaptation to age-related changes, described as “selective optimization with compensation”. Other major elements included in researcher definitions of successful ageing are: life satisfaction, longevity, freedom from disability, mastery/growth, active engagement with life, high/independent functioning, and positive adaptation (Phelan & Larson, 2002). Another review identified 29 different definitions. Most of them were based on the absence of disability, while psychosocial criteria were less common (Depp & Jeste, 2006). However, criticism has been levelled against the too narrow and excessively medical definition by Rowe and Kahn (1997), which is used in most research.

Successful ageing is a North American conceptualization developed in a certain socio-political context. The term in itself has been accused of contributing to discrimination and ageism, because it categorizes too many persons as not ageing successfully. It is believed that a multi-faceted perspective would be of greater relevance to older persons (Dillaway & Byrnes, 2009). Recent theoretical models have tried to further broaden the concept and to include both objective outcomes and subjective estimates by the individual (Bowling & Dieppe, 2005; Doyle, Mc Kee, & Sherriff, 2012; Pruchno, Wilson-Genderson, & Cartwright, 2010; Von Faber et al., 2001; Young, Frick, & Phelan, 2009). In fact, older people appear to assess themselves as successful agers more often than the researchers studying them do (Bowling, 2007; Strawbridge et al., 2002). This discrepancy has led to a greater focus on older persons’ own views of successful ageing. A few qualitative studies have been carried out—mainly in North America—(Iwamasa & Iwasaki, 2011; Knight & Ricciardelli, 2003; Reichstadt, Depp, Palinkas, Folsom, & Jeste, 2007; Reichstadt, Sengupta, Depp, Palinkas, & Jeste, 2010; Von Faber et al., 2001). According to these studies, successful ageing seems to be multi-dimensional, while adaptation, other psychological traits (optimism and sense of purpose), and social involvement are emphasized. Recent studies have not only highlighted cross-cultural similarities but also differences in perceptions of and strategies for successful ageing (Hilton, Gonzalez, Saleh, Maitoza, & Anngela-Cole, 2012; Lewis, 2010, 2011; Romo et al., 2012; Troutman, Nies, & Mavellia, 2011). To enable policy goals and strategies that are of value to the older persons themselves, successful ageing needs to be explored from the perspective of diverse groups of older people.

The present article aims to explore successful ageing from the perspective of community-dwelling older persons in Sweden.

## Methods

### Design

Qualitative content analysis is a method with the aim of providing knowledge, new insights, and a practical guide to action (Krippendorff, 2004). It is well suited to analyse data on multi-faceted phenomena and to produce conceptual models to increase understanding (Elo & Kyngas, 2008). It is considered to be a method without a solid philosophical background (Krippendorff, 2004). Initially qualitative content analyses dealt with manifest content, but over time latent content has also been included. It is an appropriate method for identifying variations in terms of similarities and differences in a text (Graneheim & Lundman, 2004). The fact that the constituents of successful ageing probably vary from person to person but also might contain something that is common and comprehensive made qualitative content analysis a suitable method for this study. A deeper knowledge of older persons’ own views on how to age successfully could guide policies, interventions, and outcome measures in a way that is more meaningful to them.

### Informants

The sample comprised 24 community-dwelling older persons in a smaller urban community in western Sweden. Their mean age was 81 years, with a range of 77–90, and their characteristics are shown in Table I.

**Table I:** Description of participants (n = 24).

Characteristics	Number or mean
Age; years, mean (range)	81 (77–90)
Female/male; n	9/15
Living alone; n	9
Need of municipal care; n	0
Contact with health care, latest 6 month; n	20
Hospitalized, latest 6 month; n	4
Self-rated health is ≥good^1^; n	20
Intervention/control; n	18/6

1 Question from Short Form-36. Response alternative on a 5-grade scale: bad, fairly good, good, very good, and excellent.

Participants were recruited from a health promotion intervention based on the concept of physical activity, which took place during 2009–2010. Inclusion criteria were ≥75 years and a low to moderate physical activity level. Exclusion criteria were living in institutions, severe health problems, and homecare (with the exception of a safety alarm and help with purchasing food). A total of 60 persons, randomized into a control group or an intervention group, were included in the original intervention, which lasted 6 months. After the intervention was completed, letters were sent and followed up by a phone call asking them to participate in interviews. Most interviewees were participants from the intervention group (n = 18). The reason for this was to also capture how they experienced the intervention, an aspect that is not included in this article. During the study we decided to also include persons from the control group (n = 6), to handle potential bias from participation in a health-promoting intervention.

### Ethical considerations

Anyone interested in participating was orally informed of the procedure, that they could at any time stop their participation, and that all data would be treated with confidentiality. The participants were given the opportunity to ask further questions and were free to choose whether the interview would be conducted in their own home or at the centre where the intervention had been carried out. Written informed consent was obtained from each participant before the interview. Approval was granted by the Regional Ethical Review Board (diary number 761-08).

### Data collection

Individual interviews were conducted by the first author with each of the participants between November 2010 and January 2011. They consisted of open-ended questions involving thoughts and experiences concerning successful ageing. The opening question was, “Please tell me what successful ageing means to you?”. The term “a good old age” was used in interviews as “successful ageing” is not an established expression in the Swedish context. Additional open-ended questions to deepen the content of the responses, in relation to the purpose of the study, were asked. A final question was also asked about how they experienced the exercise intervention, which is not presented in this article. The interviews were audio-recorded and transcribed verbatim by the first author immediately after the interview. All interviews took place in the participants’ own homes. The mean interview time was 37 min (range 12–70 min).

### Data analysis

When all participants had been interviewed, the transcribed text was analysed according to qualitative content analysis (Krippendorff, 2004). The approach as described by Graneheim and Lundman (2004) was used. The interviews were first read through several times to obtain an overall perspective of the content, focusing on the purpose of the study. An inductive approach was taken. First, sentences and phrases relevant to the issue were picked out to form meaningful units. Second, the meaning-units were condensed and coded. Third, the codes were abstracted to a higher logical level and compared for differences and similarities; first in the form of *sub-categories* and then sorted into *categories*, still close to the data and on a descriptive level (manifest content). Finally, a more abstract and interpretative *theme* was formulated from the underlying (latent) content, supposed to embrace all categories. The analysis involved reading back and forth between the whole text and the text segments. The first author analysed the data with supervision by the last author. The second author was involved in the last step of the analysis, by confirming relations between sub-categories, categories, and themes. Discussions were held until consensus was reached. A reflexive attitude, with awareness of that the perspective of the researcher shapes all research, was adopted (Malterud, 2001). The authors had no previous relation to the interviewees. The computer program NVivo 9 (QSR international Pty Ltd) was used to help organize the developed lists of codes by generating a structure of individual categories (“nodes”) and connecting codes and meaning-units to these category groups. It was also a tool in communication between the authors.

## Results

**Figure 1:**
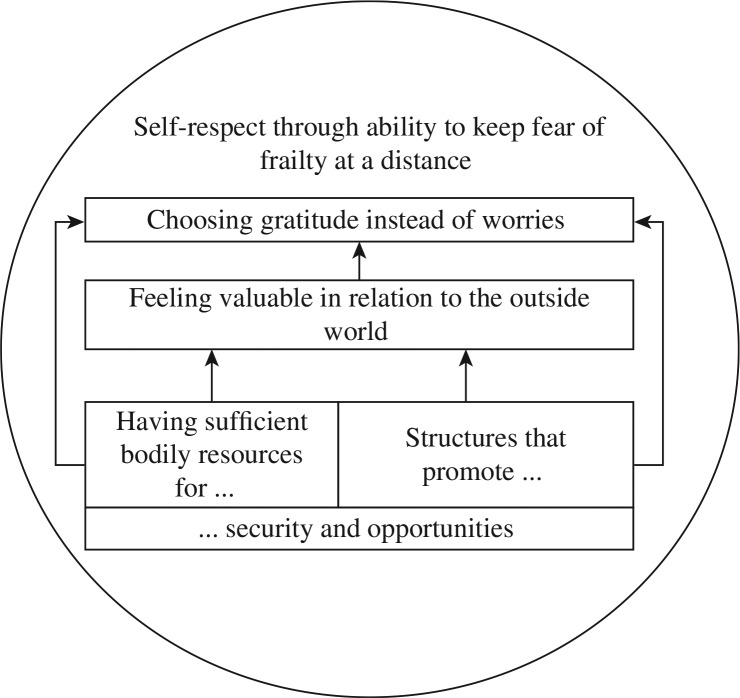
A model of successful ageing from the perspective of community-dwelling older persons.

An overarching theme of successful ageing was formulated as “self-respect through ability to keep fear of frailty at a distance”. This was achieved either by continuing to actively take part in the outside world or by trying to keep ones’ mind from focusing on oneself and one's own vulnerability. The theme embraced four categories with a somewhat interrelated and hierarchical relation; “having sufficient bodily resources for security and opportunities”, “structures that promote security and opportunities”, “feeling valuable in relation to the outside world”, and “choosing gratitude instead of worries” (Figure 1). Theme, categories and sub-categories are presented in text below and summarized in Table II.

**Table II:** Theme, categories, and sub-categories identified in analyses of successful ageing.

Theme	Category	Sub-category
Self-respect through ability to keep fear of frailty at a distance	Having sufficient bodily resources for security and opportunities	
	Structures that promote security and opportunities	Satisfaction with one's financial situation
		Security and opportunities in the closest context
		The health and well-being of close relatives and friends
	Feeling valuable in relation to the outside world	Feeling noticed and appreciated in social relations
		Engagement in activities that provide pleasure or benefit
	Choosing gratitude instead of worries	Choosing gratitude for not being as bad as others who are in a worse situation
		Denying difficulties
		Accepting things you cannot change

### Having sufficient bodily resources for security and opportunities

Health or bodily resources were often the first thing mentioned and the basis for participation in life. One was not aware of the body when everything was alright, just when there was something wrong. Health was often described as the ability to “walk on your own legs” and to take care of yourself, getting out of bed and having the energy to be active. Having good cognitive capacity and preserved personality and role-function to take part in society were also mentioned. Health was not seen as freedom from disease, but as a resource for making activity and participation on a personally desired level possible. The prevalence of medical conditions, which was very common, did not affect the experience of health. Some degree of morbidity was seen as a natural part of the ageing process, as long as independence, freedom from pain, and ability to participate on a personally desired level was preserved. Health was described as the absence of pain and limitations. Health remained silent. Major threats were pain, life-threatening diseases, being dependent on someone else, isolation, and insecurity about losing one's autonomy when close to death. An uncertainty about how life would end and not wanting to be a burden to close relatives and friends was also described.

I look after myself quite well, avoid being a hanger-on to the children, hi, hi, hi. I'm helping them in fact. I don't think it would be fun to be like an extra to them, they have quite a lot to cope with themselves you see.

### Structures that promote security and opportunities

The immediate surroundings and the closest context were described as important for the ability for successful ageing. These could either contribute to safety and opportunities, or be restrictive.

#### Satisfaction with one's financial situation

This was the factor most often mentioned. It could give stability/safety and also an extra dimension to life. The basic level was to be able to pay the bills and thus avoid worries. One participant said, “Yes, it means there are no worries, no worry about money or anything. You have everything. No worries”. Good financial circumstances could make life easier and enable a person to keep up their interests. Poor finances would prevent a person from participating in activities they enjoy, like inviting friends for dinner. Especially for widows, poor finances could be a major limitation, “Well you can't treat yourself to that little extra with the pension I have … travel, concerts and so on. It's impossible, really impossible”.

#### Security and opportunities in the closest context

The men, in particular, mentioned support from their wives in their daily life. There was also a risk of overprotection by the partner in case of medical problems. Some widows mentioned friends that regularly visited to look after and help them. When feeling down, a phone call from a friend or relative could help keep up a good mood. When they were in need of help, they expected the health care system to take care of diseases. Being informed by the health care system and knowledge concerning one's medical condition contributed to their feeling of security and health. Some of the interviewees were about to move or had just moved from their own house to an apartment. Feelings about this were mixed. Moving could be both a relief, easing a burden, and a threat, in that one could become inactive when many natural activities that they had been doing all their lives got lost. Living in a smaller community, especially if they have lived most of their lives there, was seen as an opportunity to meet people they knew. Many mentioned that being able to drive a car—or their partner being able to do so—gave a sense of freedom and made it possible for them to participate in meaningful activities. The winter season in the Scandinavian environment with ice and snow meant limitations to be active and was thereby a threat to successful ageing.

The health and well-being of close relatives and friends were almost as important as their own health. Bad health in a partner, child, or close relative was often associated with worries and sadness. To take care of a partner could be seen as a threat to their personal freedom and the opportunities for engagement in activities. The death of a spouse was an enormous threat.

### Feeling valuable in relation to the outside world

To have interests, participate in activities of various kinds, and to maintain social relations where one could feel important gave meaning to life. Threats to feelings of value included, for example, difficulty in making new friends in old age, feeling lonely by themself or among others, stopping doing things they like, and being alone and inactive.

#### Feeling noticed and appreciated in social relations

Close relatives were most important—one's spouse, children, and grandchildren. To feel loved and to care about someone else helped them to forget about themself and distracted their attention, kept them from worrying so much. Some stressed high quality in the relationship with the partner, some with children and grandchildren, and some preferred social relations with many friends. Feeling that someone liked them and that they belonged to a community, or just to say a few words to someone they recognized when out walking, could keep up their mood. In this category, preferences varied greatly in accordance with habits in earlier life. A balance between social interaction and being alone in everyday life was often desired.

Talking to other people has a positive effect I think. If you are often alone it gets a bit monotonous and isolated, like. But as a matter of fact I'm not so dependent on having folk around me all the time either. It's rather nice to be on your own too. Going for a walk, and walking properly on your own, I think is really nice actually.

#### Engagement in activities that provide pleasure or benefit

For example, cultural activities, physical activities, church, senior citizens’ associations, and to be out in the natural surroundings was a way of making life feel valuable. Many interviewees described physical and cognitive activity as the key to stay healthy. Inactivity was often seen as a threat to health as it could lead to a loss off capabilities.

Then I get going and work, you know, then I don't think about that thing, then I don't feel it in the feet, but if I were to lie down and only stay at home with the wife, ha, ha, then I would be sick, I believe that. Yes, then you feel this thing, you know, oy, oy, oy, today I can't walk … and then you just sit there.

To do something nice, make other people happy or help children or friends gave feelings of satisfaction. One participant said, “Then I've been pleased that I have something to give to others when I've been a leader on the course, I follow everybody and am interested. They think I'm good and then I'm pleased”. To do something that one is really good at or succeed after a period of practice or struggle was seen as beneficial, for example:

To spend time painting and drawing as I've always done is ever such fun. The world just disappears. And then you feel really well. That's absolutely true. I also think it helps— you are creating something. Your brains get to work, imagination, everything. It's important.

To some degree continue with your previous interests, but not at the same level as before, had the same positive effect. To gain knowledge throughout life from experience of taking part in activities/work and in social relations gave strength and confidence and a peace that they did not have earlier. If activities had to be reduced, it was important to reduce them gradually to keep one's self-respect. While many wished to continue some social interaction, they also appreciated calm and peace by themselves. A balance between variation and structure and also between activity and rest was preferred.

### Choosing gratitude instead of worries

Strategies to deal with an increased vulnerability in relation to one's own body and one's immediate surroundings were seen as necessary for successful ageing. All participants had to cope with stressors of various kinds, for example, the death of close friends or relatives, loss of their own capacities, and the presence of diseases. This was described as a conscious choice, something that was possible to be in control of. This was a constant struggle for those with most limitations. However, many experienced that they had an inner strength to deal with stressors in life when needed. They were able to keep on focusing on things they could influence and not feel sorry for themselves. This strategy could be reinforced if they were noticed and loved by other people. A focus on losses and worries about the future or regrets in the past was considered to be a threat.

#### Choosing gratitude for not being as bad as others who are in a worse situation

Most interviewees felt grateful for their situation, irrespective of personal health problems, by comparing themselves with others in worse situations, expressed for example as:

Being sufficiently in order every morning you have to get up that you can get up out of bed. You see an awful lot of people who come in their wheelchairs and with assistive devices.

Or

Then you also have to realize that not everyone can have the advantage of being able to keep mobile till you are in your 78th year. You also have to think that there are some who have it much, much worse. You have to think that. You must not be too egoistic. That I should be able to do this and I should have that. No, you shouldn't. But it's easy to do, easy to do.

This applied not only to persons of similar age but also to their own parents and the way they aged, and even to their own situation when they were young.

#### Denying difficulties

A focus on capacities and not on worries or regrets seemed necessary. Denial of difficulties was the only way to go. Never stop trying. Keep doing things, but in a different way. Many of the interviewees laughed when they mentioned death or illness. That might be an expression of trying to keep a distance from stressors in life. One participant said:

Yes, but then it's a question of shutting out those thoughts, quick as hell. You must do that, because you can't go around fretting all the time that you can't and can't do that. That would be awful (laughs). Think of something else. It works well. And then, make a fresh start and try, and do what you can.

#### Accepting things you cannot change

To accept what you cannot influence, both your own limitations and the fact that relatives and friends are ill or no longer alive, was described as necessary. Accept life the way it is, and that it includes both sorrows and happiness. This was seen as a wisdom that could be learnt throughout life. One participant said:

What you cannot do anything about, you have to go through. One should keep spirits up yet somehow. Most things have gone well, sorrows and diseases. But it is clear that it wears out … but if you can argue that it is what belongs to life so it may take its course. I think it's so … for me. It has been so in all cases. It's nice.

Some of the interviewees emphasized that having a religion and belonging to a church community could be a support in managing age-related changes and losses and a way to find consolation. One participant said, “I think that being a Christian has helped me, given me great comfort”.

## Discussion

Our article provides evidence that a preserved self-respect through ability to keep fear of frailty at a distance is of importance for successful ageing. To be seen and confirmed by others in a positive way was also highlighted. This was achieved either by active involvement or by strategies to focus the mind outside the self. Societal attitudes towards older persons seem to have an impact on the opportunities for successful ageing, by increasing or decreasing the possibilities for preserved value and self-respect. Self-respect is most often seen as a self-reflection of the recognition and appraisal we receive from others. Another dimension of respect is status self-respect, that is, recognition of our place in society, dealing with our sense of autonomy, mastery, and our place in society (Middleton, 2006). This aspect is probably of particular relevance for older persons in many societies of today where youth and independence is the norm. To preserve one's self-respect, too much focus on oneself was not desirable and the ability and conditions for keeping involved in other persons or interests were emphasized. Attention has been drawn to this struggle to preserve one's identity as one of the processes connected with growing old (Fischer, Norberg, & Lundman, 2008). According to Eriksson and Eriksson's (1997) developmental theory, a life-course perspective on identity seems needed to reach an ability to adapt to losses and stressors in old age. If there is a successful development of personality during different stages of life, wisdom rather than predominantly regrets or despair could be the result. However, in our findings, worries for increased vulnerability in the future, and not regrets for the past, were the dominating threat to feelings of value. Eriksson and Eriksson (1997) also emphasize the interaction of body, mind, and culture in this process. Our findings are in line with research suggesting that successful ageing from the perspective of older persons seems to be a continuum and involve multi-dimensional aspects like one's own bodily resources, the closest context, meaningful engagement with life, and a process of adaptation (Reichstadt et al., 2007, 2010).

The finding “Having sufficient bodily resources for security and opportunities”, could be seen as confirming the predominant biomedical definition of successful ageing by Rowe and Kahn (1997). However, our findings suggest that absence of disease seems almost impossible and irrelevant as long as independence and ability for involvement is preserved. The emphasis on health and independence could be a reflection of societal values in western countries, where independence is a norm. It might not be as closely connected to success in all cultures. Although, the meaning of independence in our findings was close to those of Japanese Americans who have been shown to emphasize not wanting to trouble others as means to meet the own needs (Iwamasa & Iwasaki, 2011). The fear of being dependent was seen as a threat to successful ageing. According to the widely accepted biomedical definition of successful aging, a period in life where one is dependent on another person could not be successful. This period is sometimes referred to as the fourth age, as opposed to the third age, which is described as a period after working life where one is still healthy and able to fulfill personal goals (Laslett, 1991). Therefore, the struggle for independence and involvement as described by our interviewees may in situations of dependence have to be exchanged for acceptance of being part of a whole and of the world, from being a subject to being an object (Fischer, Lundman, & Norberg, 2010). As opposed to the perceptions of the rather healthy older persons in our study, older persons at the end of life with bodily restrictions and in need of municipal care have been identified to use a turning inwards and a reflective attitude when trapped by health complaints (Andersson, Hallberg, & Edberg, 2008). This stage is closely related to the disengagement theory (Cummings & Henry, 1961) and gerotranscendence (Tornstam, 2005). The process of separation from societal roles might not be possible or desirable before the body reaches this stage, that is, embodied knowledge.

We also found that external factors, especially economy, were of importance to avoid worries and enable involvement. Structural factors on a societal level have been suggested as an explanation for cross-national variations in successful ageing in Europe (Hank, 2011) and for disparities in successful ageing between socially defined groups in the United States (McLaughlin, Connell, Heeringa, Li, & Roberts, 2010). Structural factors have also been identified as means of security and stability in qualitative interviews with older persons (Reichstadt et al., 2007). However, this aspect has only recently been included in multi-dimensional models (Iwamasa & Iwasaki, 2011). On contrary, structural aspects are included in multi-dimensional models of quality of life in old age (Mollenkopf & Walker, 2007). Another external factor, not identified in previous research, was the climate, in this case the Scandinavian winter, which seemed to influence the possibility for involvement and participation in a negative direction.

To continue with social activities and interests on a personally desired level, as long as possible, were seen as important as providing opportunities to feel valuable, loved, confirmed, and significant for someone else, and as a distraction from worries. This finding emphasizes the importance of confirmation and the strengthening of a positive self-identification. Others have identified feelings of usefulness as important for successful ageing and it has been proposed to predict mortality and shape health trajectories in old age (Gruenewald, Karlamangla, Greendale, Singer, & Seeman, 2007). The way many of the interviewees in our study used to achieve value and importance supports the continuity theory (Atchley, 1989), which implies that older adults from a life-course perspective usually maintain activities by using adaptive strategies. The continuity theory is elaborated and modified from the activity theory (Havighurst, 1961), which implies that maintenance of engagement and activity is essential in that one's self-image is preserved. Others have also stressed that staying physically and mentally active are important for a meaningful life in this age group (Doyle et al., 2012; Gunnarsson, 2009).

To actively choose gratitude and to keep worries at a distance was seen as necessary for successful ageing. This was described by some interviewees as an inner strength that came naturally. This might indicate that a life-course perspective of how people cope with stressors is of relevance. Adaptation and a positive attitude are in line with the psycho-social theory of selection, optimization, and compensation (Baltes & Baltes, 1990). In our findings, comparison with others in a worse situation was a common strategy for coping with increased vulnerability. This strategy made gratitude possible despite increased prevalence of stressors in life. Older persons with a high quality of life have previously been shown to use this strategy, while frail elderly with a low quality of life compared themselves with people in a better situation (Puts et al., 2007). Downward social comparison has been suggested to be a way of avoiding a focus on regrets and a pathway to successful ageing (Bauer, Wrosch, & Jobin, 2008). Avoidance of focus on bad things, or even denial of difficulties was in our findings the main strategy. It could be argued that denial is not a good method to achieve well-being. However, if there is no chance of influencing or mastering a situation, which is often the case concerning stressors in old age, it could be a proper way to cope (Lazarus & Lazarus, 2006).

## Methodological considerations and limitations

To validate the study findings for trustworthiness; credibility, transferability, dependability, and confirmability were considered (Graneheim & Lundman, 2004; Krippendorff, 2004; Lincoln & Guba, 1985).

Credibility, that is, the confidence in the “truth” of the findings, was addressed by including a strategic sample according to gender and living conditions, and including persons from both the intervention and the control group, and choosing a number of interviewees to ensure variation. Further, the first and the last authors compared codes, sub-categories, and categories throughout the analysis. With reference to professional experience, all authors were physiotherapists. The first author had previously worked for 10 years in hospital care, the second author for 20 years in hospital care, and the third author for 10 years in primary care. However, the interviewees were only informed about the occupation of the interviewer when they asked for it. Peer debriefing by persons with different backgrounds and experience was used as a way to explore new aspects of the phenomenon. Links between the data and results are illustrated in quotes in running text.

For the reader to evaluate transferability, that is, the possibility of transferring the results to other contexts, a clear description of the interviewees and their context, data collection, and process of analysis is given.

Dependability and confirmability, that is, the extent to which the same findings will appear under similar circumstances and how well the results can be confirmed by others, were addressed by following the same procedure in every interview and by including dialogue among co-researchers with different professional backgrounds. A reflexive attitude was adopted and the pre-understanding by the first author was recorded before starting the interviews and was continuously reflected upon (Malterud, 2001). Raw data and the analytical procedure were documented in NVivo.

There are several limitations to consider. First, the interviewees were collected from a randomized controlled trial. This reduced the possibility for strategic selection and led to a more homogeneous sample. The fact that most interviewees had been participating in a health-promoting intervention could also have biased their answers in favour of a more positive attitude and a stronger emphasis on activity in general and physical activity in particular. Second, all interviewees were from a rural area in Sweden and the perspective of older people from urban areas could differ. Third, the length of the interviews ranged from 12 to 70 min, indicating that persons who had the easiest to express themselves and reflect over their situation contributed the most to the results. Fourth, the interviewees comprised mostly men. One reason for this was that it was difficult to recruit women to participate in the original intervention. Some gender differences were identified, and it could be interesting to analyse women and men separately. Gender differences have been described for many aspects of ageing and, despite their lower life expectancy, men have been shown to have a higher prevalence of “success” and health as compared to women (Hank, 2011). This difference also appears to become larger with age (Thielke & Dier, 2012). Fifth, the word *success* could have different meanings in different cultures which are non-English speaking. In Europe, the concept of *healthy ageing* is more common than *successful ageing*, and in Sweden *ageing well* is also often used. To choose the expression *a good old age* in interviews could have affected answers in that less emphasis was laid on positive aspects only. Despite the concept selected, quality of life, successful ageing, healthy ageing, and other positive conceptualization seem to refer to the same interest in maintaining a good life at old age, and we should focus on what is common to these concepts rather than differences between them. However, one always has to consider the risk of ageism. Sixth, all the researchers had the same original profession, namely physiotherapists, and a multi-disciplinary team would have been preferable. Finally, the interviewees in this study were born during the 1930s or late 1920s. Their historicity might contribute to feelings of gratitude and lower expectations, compared to cohorts born later with higher expectations due to more advantaged conditions during childhood.

## Conclusion

Successful ageing can be seen as a preserved self-respect through ability to keep fear of frailty at a distance. It seems to be a dynamic process rather than a static structure and might therefore be susceptible to actions (Lazarus & Lazarus, 2006). Paying attention to and respecting worries regarding an increasing vulnerability, while at the same time focusing on older persons’ abilities and strategies for preserving their self-respect, can lead to better ways of promoting successful ageing in those still living in the community. This highlights the importance of societal attitudes towards ageing, which are reflected in older persons.
